# Can a core outcome set improve the quality of systematic reviews? – a survey of the Co-ordinating Editors of Cochrane review groups

**DOI:** 10.1186/1745-6215-14-21

**Published:** 2013-01-22

**Authors:** Jamie J Kirkham, Elizabeth Gargon, Mike Clarke, Paula R Williamson

**Affiliations:** 1 Department of Biostatistics, University of Liverpool, Shelley’s Cottage, Brownlow Street, Liverpool, L69 3GS, UK; 2 Centre for Public Health, Queen’s University Belfast, Grosvenor Road, Royal Victoria Hospital, Belfast, BT12 6BA, UK

**Keywords:** COMET, Cochrane review groups, Co-ordinating editors, Core outcome set, ORBIT, Systematic reviews, Survey

## Abstract

**Background:**

Missing outcome data or the inconsistent reporting of outcome data in clinical research can affect the quality of evidence within a systematic review. A potential solution is an agreed standardized set of outcomes known as a core outcome set (COS) to be measured in all studies for a specific condition. We investigated the amount of missing patient data for primary outcomes in Cochrane systematic reviews, and surveyed the Co-ordinating Editors of Cochrane Review Groups (CRGs) on issues related to the standardization of outcomes in their CRG’s reviews. These groups are responsible for the more than 7,000 protocols and full versions of Cochrane Reviews that are currently available, and the several hundred new reviews published each year, presenting the world’s largest collection of standardized systematic reviews in health care.

**Methods:**

Using an unselected cohort of Cochrane Reviews, we calculated and presented the percentage of missing patient data for the primary outcome measure chosen for each review published by each CRG. We also surveyed the CRG Co-ordinating Editors to see what their policies are with regards to outcome selection and outcomes to include in the Summary of Finding (SoF) tables in their Cochrane Reviews. They were also asked to list the main advantages and challenges of standardizing outcomes across all reviews within their CRG.

**Results:**

In one fifth of the 283 reviews in the sample, more than 50% of the patient data for the primary outcome was missing. Responses to the survey were received from 90% of Co-ordinating Editors. Thirty-six percent of CRGs have a centralized policy regarding which outcomes to include in the SoF table and 73% of Co-ordinating Editors thought that a COS for effectiveness trials should be used routinely for a SoF table.

**Conclusions:**

The reliability of systematic reviews, in particular meta-analyses they contain, can be improved if more attention is paid to missing outcome data. The availability of COSs for specific health conditions might help with this and the concept has support from the majority of Co-ordinating Editors in CRGs.

## Background

Cochrane Reviews are systematic reviews of research in human health care and health policy, and are internationally recognized as a high quality source of evidence for decision making. They bring together research evidence on the effects of healthcare interventions or the accuracy of diagnostic tests into the world’s largest, standardized collection of systematic reviews, with more than 5,000 full reviews and protocols for another 2,000 published to date. Outcome selection in systematic reviews needs to be relevant to patients, clinicians and policy-makers if the findings of a review are to influence practice and future research. Inconsistent choice of outcome measures in clinical trials means that many meta-analyses are unable to include data from all the relevant studies. For example, the five most accessed Cochrane Reviews in 2009, together with the top cited review in that year, all described inconsistencies in the outcomes reported in eligible trials, which hampered the ability of the reviewers to resolve the uncertainties about health care that they set out to tackle [1]. An additional problem that can affect the quality of evidence within a systematic review is that of missing outcome data from all eligible trials. Missing outcome data in trials can occur for a number of reasons. Participant data can be missing as a result of attrition or as a result of the selective non-reporting of an outcome in a study. Recent research has shown that outcome reporting bias (ORB), that is, results-based selection for publication of a subset of the original outcome variables, is also a major problem in randomized trials [2]. The Outcome Reporting Bias in Trials (ORBIT) study of the impact of ORB in randomized trials on the results of Cochrane Reviews found that ORB is an ‘under-recognised problem that affects the conclusions in a substantial proportion of Cochrane reviews’ [3]. This study found that more than half (157/283 (55%)) of the reviews did not include full data for the review’s primary outcome of interest from all eligible trials. The median amount of review primary outcome data missing for any reason was 10%, whereas 50% or more of the potential data were missing in 70 (25%) reviews. In this same study, an assessment of the impact of ORB was undertaken for reviews with a single meta-analysis of the primary review outcome where there was missing outcome data for a whole study that was known or suspected of being measured but not reported. Of the 25 reviews that met these criteria, a sensitivity analysis revealed that the conclusions to eight of these reviews were not robust to ORB—that is, the treatment effect estimate changed from a significant result favoring treatment to a non-significant result. For all the 25 reviews assessed, the median percentage change in the treatment effect estimates after the adjustment based on the sensitivity analysis was 39% (interquartile range 18% to 67%) [3].

One way to reduce the amount of missing outcome data by ensuring that eligible trials contribute usable information is the definition and implementation of an agreed minimum set of standardized outcomes, to be measured and reported in all trials for a particular disease or condition, referred to as a ‘Core Outcome Set’ (COS) [4,5]. Furthermore, some Cochrane Review Groups (CRGs) now include a Summary of Findings (SoF) table in their reviews [6]. The SoF table presents the results of the review for up to seven outcomes that are important to patients and aims to improve the understanding and retrieval of key findings.

In this paper, we present results for missing data in single review primary outcomes from the Cochrane Reviews included in the ORBIT study. We also report the results of a survey of the Co-ordinating Editors of CRGs, undertaken to obtain their views on the benefits and challenges of core outcome sets and how they might improve the quality of the reviews in their CRG. We were also interested in establishing whether the amount of missing data from a systematic review was influenced by whether or not a CRG had a centralized policy on outcome selection. The Co-ordinating Editors have responsibility for overseeing the work of their CRG and deciding on the publication of each of the Cochrane Reviews from within their CRG.

## Methods

### Missing data

The missing study data reported here was part of the larger ORBIT project to estimate the prevalence and effect of outcome reporting bias in clinical trials on the primary outcomes of systematic reviews from three issues of The Cochrane Library: issue 4, 2006; issue 1, 2007, and issue 2, 2007. For the 283 reviews in this study, the number of participants in each of the eligible studies was extracted and the percentage of missing participant data was calculated as the number of participants missing from the meta-analyses of the single review primary outcome (as a result of the non-reporting of outcome data in a study), as defined by the lead reviewer. Data from reviews containing at least five eligible studies are presented.

### Survey of Co-ordinating Editors of Cochrane Review Groups

With the exception of the Cochrane Methodology Review Group (which focuses on topics relating to methodology, rather than healthcare interventions and tests), the Co-ordinating Editors for the 50 CRGs (as of 2011) were contacted and asked to participate in a short online survey with regards to how a COS might improve the quality of the reviews in their CRG. The survey was constructed using the online survey software, SurveyGizmo, and was open for responses between 1 November 2011 and 31 July 2012. Non-responders were contacted periodically if a response to the survey was not received. As part of the survey, Co-ordinating Editors were provided with a brief synopsis of how COSs could make it easier for their review authors to identify appropriate outcomes for Cochrane Reviews, and present their findings clearly and succinctly. They were also presented with the missing data plot for all CRGs and made aware of the data relating to their CRG. Co-ordinating Editors were asked whether or not they had any centralized CRG policies relating to the outcomes to include in their reviews (including the distinction between primary and secondary outcomes) and outcomes to include in the SoF tables (providing one is included in the review), or whether these decisions are left to the authors of the review. Patterns in the amount of missing participant data between CRGs with and without a centralized policy on outcome selection were examined. Finally, Co-ordinating Editors were asked if they thought that a COS for effectiveness trials should be used routinely for a SoF table and what they thought were the main advantages and challenges of standardizing outcomes across all reviews in a particular condition covered by their CRG. All advantages and challenges were independently reviewed by two authors (JJK and PRW) and categorized into common topics. Any discrepancies were resolved through discussion.

## Results

**Figure 1:**
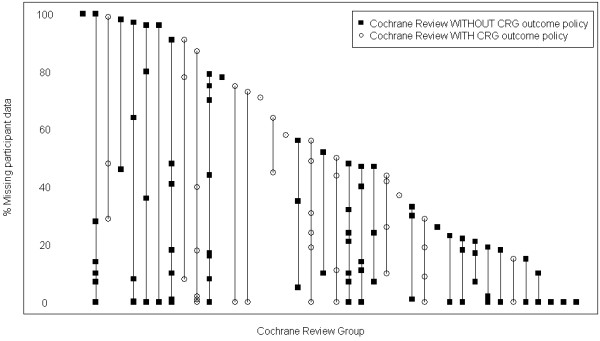
The percentage of missing patient data for the primary outcome for each review that had at least five included studies (reviews first published in Issue 4, 2006 to Issue 2, 2007).

Of the 283 reviews included in the ORBIT study, 143, from 40 CRGs, were included here, that is, reviews that contained at least 5 eligible studies (Figure 1). Forty-one (29%) reviews contained no missing patient data, while 26 (18%) had more than 50% of the patient participant data on the single primary outcome missing from the review. Accountable reasons for missing data included the difficulty to measure certain outcomes, the diverse range of possible outcomes to measure and disputes around the importance and best outcomes to measure which are both robust and reliable for use in systematic reviews. A summary of the relevant responses with regards to the reasons for missing outcome data that was provided by the Co-ordinating Editors is provided in Table 1.

**Table 1:** Responses from Cochrane review group co-editors who provided reasons for missing data in their reviews

**Cochrane Review Group**	**Explanation from Cochrane Review Group Co-Editor**
Pain, Palliative Care and Support Care	‘I think this is about right [amount of missing data]. Our scope is large and these problems are more common when there are no received methods (e.g. rehabilitation, palliative care, complex interventions) and less common where there is a history of methods development and consensus (e.g., headache).
Dementia and Cognitive Impairment	‘Would suggest that it may not be possible in our CRG to specify outcomes only by the condition without reference to the type of intervention. For example, some interventions in dementia may aim to modify the course of the disease, and cognitive outcomes should clearly be part of a minimum outcome set in such trials; for others, such as certain psychosocial interventions (e.g. touch and massage), modifying cognitive decline would be an unreasonable aim, and collection of cognitive data would be unduly burdensome for patients and researchers’.
Oral Health	‘Our reviews are very diverse so we would need to develop many different core outcome groups’.
Neuromuscular Diseases	‘Neuromuscular Disease is very diverse, even sometimes within one disease. For instance some patients with Motor Neuron Disease will have lots of spasticity and complications related to that, others flaccidity and others bulbar involvement. Variations in outcomes chosen depend upon the focus of the intervention and the researcher. We try to keep our outcomes fairly loosely defined - e.g. any measure of walking ability - especially in physio/fatigue/strength training and other qualitative research areas - but even then there are some fairly abstract outcomes measured that don’t fit in clearly’.
Renal	‘The problem is the heterogeneity of our scope. Unlike some other CRGs we are an organ-related CRG (and then cover many orphan reviews). We cover everything from UTI (urinary tract infections) to transplantation’.
Cystic Fibrosis and Genetic Disorders	‘One set of outcomes is not always appropriate for every trial in cystic fibrosis, e.g. for a nutritional intervention body mass index may be appropriate. For a respiratory therapy it may be spirometry’.
Heart	‘The issue of missing data for primary outcomes may reflect the biomedical bias in many heart disease trials - so if we have 20 RCTs in a review with a clinical primary outcome specified, it is very likely that only a few of the trials will have reported on the clinical outcome but many more will have reported on physiological outcomes..Our scope is much wider than many review groups, so I can envisage difficulties coming up with a core outcome set’.
Wounds	‘Our reviews cover a variety of conditions and interventions (probably most groups do) and relate to topics with quite under-developed research cultures (nursing, dermatology, surgery) so the high levels of missing data in our field doesn’t surprise me’.

Responses to the survey were received from Co-ordinating Editors for 45 (90%) of the 50 CRGs. Fourteen Co-ordinating Editors (31%) had previously been involved in the development of a COS and 16 (36%) were aware of other work that has been done or is ongoing to develop a COS for conditions relevant to their CRG. Overall, 16 (36%) CRGs have a centralized policy for outcome selection while the remaining 29 leave outcome selection and the relative importance of outcomes chosen to the discretion of the authors of the relevant review. From Figure 1, there did not appear to be any association between the existence of a CRG policy for outcome selection and the amount of missing data in their reviews. Similarly, 16 (36%) CRGs have a centralized policy regarding which outcomes to include in the SoF table (14 of which had a centralized policy for outcome selection), while 25 (56%) left this decision to the review authors and a further four (9%) are not yet including the SoF table in their reviews. All four CRGs where the SoF has not yet been adopted had no CRG policy for outcome selection but two of the four Co-ordinating Editors were aware of ongoing COS development work for conditions covered by their CRG.

A total of 33 (73%) Co-ordinating Editors thought that a COS for effectiveness trials should be used routinely for a SoF table. Reasons for this were provided by 23 Co-ordinating Editors and included relevant outcomes measured and reported (10), comparability of outcomes (6), improved interpretability of outcomes (4), standardization of outcomes (2) and reduces risk of bias (1). Ten of the 12 Co-ordinating Editors who did not think a COS should be used in the SoF table thought that the scope and diversity of their reviews would not allow a COS to be applied across the reviews for their CRG.

The Co-ordinating Editors listed 100 advantages and 82 challenges associated with standardizing outcomes across reviews in their CRG (Table 2). The most common advantage for having a COS was that systematic reviews or meta-analyses would benefit from the standardization of outcomes. The main challenges related to the process needed to develop a COS, decisions around when a COS should be applied and the need to persuade potential authors of Cochrane Reviews to use the COS.

**Table 2:** Advantages and challenges of standardizing outcomes across all reviews for a particular condition

**Advantages**	**n**	**Challenges**	**n**
Advantage for a systematic review/meta-analysis	39	Development of a COS	23
Improves interpretation/guidance	19	Something about scope	21
Outcome likely to be more appropriate	16	How to persuade authors/trialists/industry to implement	20
Advantage for the design of a new study	13	‘How’ to measure once the ‘what’ has been decided	11
Improves something about the outcome itself	6	Important outcomes not currently being measured	2
Reduces outcome reporting bias	6	Resource to develop	2
Reduces resource requirement	1	Updating process	2
		Conflict of interest	1

## Discussion

There is considerable variability within and between CRGs in the amount of data that are missing for the primary outcomes chosen for a review. However, there did not appear to be any association between the existence of a CRG policy for outcome selection and the amount of missing data in their reviews (Figure 1). Furthermore, the survey of Co-ordinating Editors revealed a clear lack of clarity and consensus on the outcomes to measure within the specialties covered by CRGs.

The set of reviews that are reported in this study are slightly dated. However, the reviews used came from the earlier ORBIT study which started in 2006. The missing participant data from the primary outcome from each review was extracted during the ORBIT study, although this remained unpublished data. Presenting the data here accompanied the Cochrane Co-Editor survey data well and demonstrated the importance of COSs from a reviewer perspective. A recent inspection of a random sample of 20 of the 283 reviews used in ORBIT revealed that only 25% (5/20) of these reviews identified new studies for inclusion in the most updated version of the review – this meant on average, in at least 75% of cases, the percent missing data from the reviews used in 2006/2007 still remains unchanged.

The suggestion that a COS should be routinely used as part of a SoF table was well received, with most Co-ordinating Editors believing that this would be beneficial. The scope and diversity of reviews within some CRGs is likely to be a barrier to the application of a COS across the reviews for the particular group. It was noted by some of the Co-ordinating Editors in these CRGs that outcome specification for particular conditions would be difficult without further refinement to specific populations and interventions (for example, pharmacological versus non-pharmacological treatments).

The main advantage of a COS as suggested by the majority of Co-ordinating Editors was that the concept of a COS would benefit the systematic review process by increasing the amount of usable information for use in a meta-analysis. The challenges that were identified appear to reflect a lack of experience with COS, which could be overcome with training or support materials (for example on how to develop a COS and how to encourage others to use it). To help with this, the Core Outcome Measures in Effectiveness Trials (COMET) Initiative brings together researchers interested in the development and application of COS (http://www.comet-initiative.org). COMET aims to collate and stimulate relevant resources (both applied and methodological), facilitate the exchange of ideas and information, and foster methodological research. The Initiative has three strategic goals: to increase the number of COSs developed using evidence-based methods; to increase their impact on the quality of research by raising awareness and increasing their use; and to establish methods for the development of COS [5]. This will include work with Co-ordinating Editors and others in The Cochrane Collaboration to facilitate activities within CRGs to develop COS and to implement these within Cochrane Reviews, including the provision of training workshops and the preparation of training materials. This work should also have benefits for systematic reviewers outside of The Cochrane Collaboration, who wish to maximize the impact of COS on the quality of reviews and the research studies that they bring together.

## Conclusions

The reliability of systematic reviews, in particular meta-analyses they contain, can be improved if more attention is paid to missing outcome data. The availability of COSs for specific health conditions might help with this and the concept has support from the majority of Co-ordinating Editors in CRGs.

## Abbreviations

COS: core outcome set; CRG: Cochrane Review Group; ORB: outcome reporting bias; ORBIT: Outcome Reporting Bias in Trials; SoF: summary of findings.

## Competing interests

PRW chairs the Management Group of the COMET Initiative. MC is also a member of the COMET Management Group and EG is the COMET Project Co-ordinator. JJK has no conflict of interest to declare.

## Authors’ contributions

JJK, EG, MC and PRW designed the survey of co-editors. The electronic version of the survey was set up by JJK. The amount of missing data from each review was extracted by JJK, who produced the missing data plots for each CRG. JJK, EG and MC sent invitations and reminders to Co-ordinating Editors to complete the survey. JJK prepared the initial manuscript. PRW and MC were involved in the revision of this manuscript. PRW conceived the idea for the study. All authors commented on and approved the final manuscript before submission.
